# Salvage procedures in lower-extremity trauma in a child with hereditary motor and sensory neuropathy type I: a case report

**DOI:** 10.1186/1752-1947-6-276

**Published:** 2012-09-04

**Authors:** Martin Gothner, Marcel Dudda, Thomas A Schildhauer, Thomas Klapperich

**Affiliations:** 1 Department of General and Trauma Surgery, BG-University Hospital Bergmannsheil, Ruhr-University Bochum, Bürkle de-la-Camp Platz 1, Bochum, 44789, Germany; 2 Department of Orthopaedic Surgery, Marienhospital Papenburg-Aschendorf, Am Hauptkanal rechts 75, Papenburg, 26871, Germany

**Keywords:** Open tibia fracture, Childhood, Neuropathy, Llizarov or Taylor spatial frame, Non-union, Infection

## Abstract

**Introduction:**

Fractures of the lower extremity are a common type of childhood injury and many can be treated without surgery. Dislocated and open fractures are an indication for fracture stabilization via either intramedullary nailing or, in the case of complicated fractures, external fixation. But if complications are likely because of diseases and disabilities (for example, a neuropathy) that can complicate the post-operative procedure and rehabilitation, what options does one have?

**Case presentation:**

We report a nine-year-old Caucasian girl who had hereditary motor and sensory neuropathy type I and who was admitted with a grade I open tibia fracture after a fall from a small height. Plain radiographs showed a dislocated tibia and fibula fracture. An open reduction with internal fixation with a compression plate osteosynthesis was performed, and soft tissue debridement combined with an external fixateur was undertaken. Three months later, she was re-admitted with localized swelling and signs of a local soft tissue infection in the middle of her tibia. Plain radiographs showed a non-union of the tibia fracture, and microbiological analysis confirmed a wound infection with cefuroxime-sensitive *Staphylococcus aureus*. Because of the non-union, the osteosynthesis was replaced with an Ilizarov external fixateur, and appropriate antibiotic therapy was initiated. Four months after the initial accident, the fracture was consolidated and we removed the external fixateur.

**Conclusions:**

If there is a pre-existing neuropathy and if disease makes it difficult for a child to follow all post-operative instructions, salvage procedures should be kept in mind in case of complications. There are multiple therapeutic options, including osteosynthesis, intramedullary nailing systems, cast therapy, or an external fixateur like the Ilizarov or Taylor spatial frame system. The initial use of an external fixateur such as an Ilizarov or Taylor spatial frame in patients with pre-existing neuropathies should be kept in mind as a possible treatment option in complicated fractures, especially in a child with pre-existing neurological or endocrine pathologies.

## Introduction

Fractures of the lower extremities are a common injury in childhood, and isolated fracture of the tibia is the most common injury caused by trauma [1]. In 70% of all injuries of the lower extremity in childhood, an isolated tibia fracture can be found, whereas both the tibia and fibula are involved in only 30% of cases [1]. Most cases involve an oblique fracture; a greenstick fracture of the tibia is a rare finding and occurs in only 10% of all cases [1]. Tibia fractures often cause a temporary disturbance of growth and a stimulation of the epiphyseal cartilage, which may result in a mismatch in growth [2]. Closed tibia fractures with minimal dislocation can be treated without surgery [3]. Dislocated and open fractures, injuries resulting in compartment syndrome or damage to the surrounding neurovascular structures, are an indication for surgery [4]. The treatment of choice for tibia fractures in childhood is closed reduction and internal fixation with intramedullary nailing or, in cases of open and complicated fractures, external fixation [5].

The open tibia fracture is an orthopedic emergency necessitating immediate surgery. Multiple treatment options for open fractures exist. In the case of grade I open fractures, internal fixation and coverage of the tissue defect can be performed, whereas the treatment for grade III fractures with a significant segmental defect via internal fixation and early soft tissue reconstruction may be associated with multiple complications, including non-union and infection; in these cases, primary stabilization with external fixation by means of an Ilizarov or Taylor spatial frame (TSF) external fixateur with soft tissue coverage could be the option of choice [4,6].

The hereditary motor and sensory neuropathies (HSMNs) are genetically heterogeneous and have autosomal dominant, autosomal recessive, and X-linked dominant patterns of inheritance [7]. All three forms are characterized by various patterns of demyelination and/or axonal atrophy of motor and sensory neurons [7]. This disease has been previously divided into three types: HSMN type I, characterized by slow conduction velocities and demyelinating nerve changes with onset during early childhood; HSMN type II, with normal or slightly reduced conduction velocities, axonal nerve pathology, and an onset in the second decade of life; and X-linked dominant HSMN, which is associated with atrophy and weakness of the foot muscles and which presents in the first or second decade of life. This peripheral neuropathy may be accompanied by a cavovarus foot, poor foot position, and difficulty bearing weight.

All types of HSMN are usually associated with peroneal and distal leg muscle wasting and weakness as well as distal sensory loss and areflexia [7]. Motor and sensory nerve conduction velocities are reduced following the loss of nerve fibers and/or demyelination. Sensory symptoms or pain is uncommon, and vibration and position sense are usually lost distally. The severity of the signs and symptoms may differ among affected individuals. Ankle reflexes are absent in nearly all patients. In type I patients, sensory and reflex changes usually are distal and are demonstrable by electrophysiological examination. Because of the peripheral neuropathy and poor motor control of the foot, patients normally find it difficult to stand still and they shift their legs constantly because of the need to maintain balance [8]. The sensory loss and the constant movement of the lower extremity can lead to foot and ankle deformities and injuries, which because of reduced pain sensation are not noticed by the patients.

The HSMN types can be distinguished by deoxyribonucleic acid analysis and electrophysiological examination as well as by the typical clinical findings. A nerve biopsy and pathological findings – such as the loss of larger myelinated fibers, the accumulation of degenerating mitochondria, the presence of dense bodies, the condensation of neurofilaments, the presence of myelin figures, and the formation of adaxonal invaginations – can help to confirm the disease [8]. To date, no therapeutic strategies for this disease have been established. A physiotherapeutic approach seems to be essential to avoid foot and ankle deformities.

Internal fixation with intramedullary nailing and soft tissue coverage is the treatment of choice for grade I open tibia fractures in the pediatric population [5]. The post-operative treatment depends on consequent wound control, physiotherapeutic mobilization, and a compliant patient. But what happens if the patient is not able to follow all post-operative instructions and complications are pre-programmed? A complicated fracture of the lower extremity combined with a neuropathy often needs a well-thought-out therapeutic strategy, and salvage procedures should be thought out in advance in case of complications.

## Case presentation

**Figure 1:**
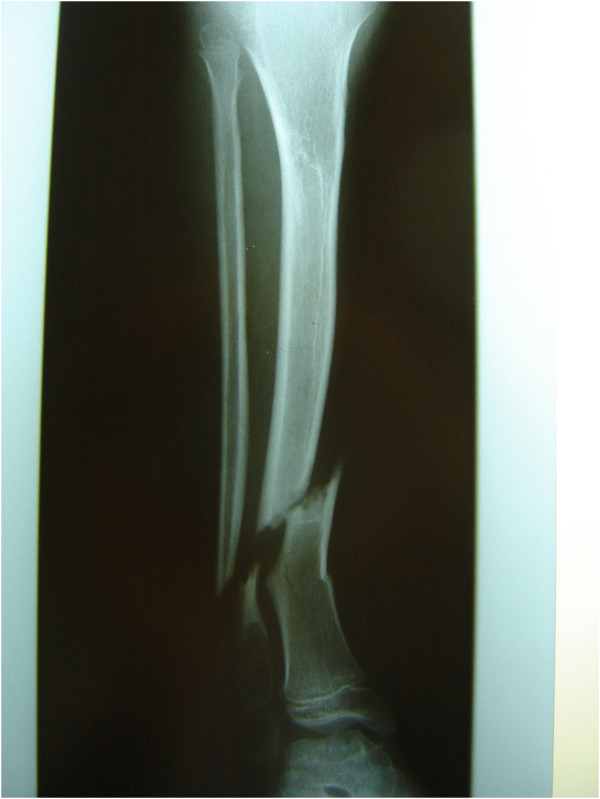
Grade I dislocated open fracture of the lower extremity (anterior-posterior).

**Figure 2:**
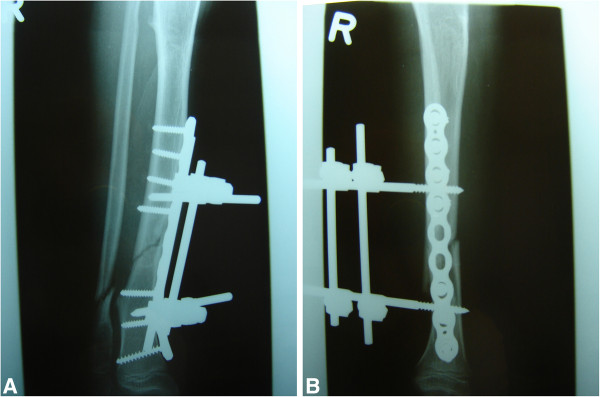
(a,b) Post-operative plain radiograph of the lower extremity after osteosynthesis and application of an external fixateur (anterior-posterior and axial).

We report a nine-year-old Caucasian girl with HSMN type I diagnosed in early childhood with resulting pain and complete loss of the distal sensory system. She was unable to stand still and constantly shifted her lower extremities to maintain her balance. In the past, she had been admitted at the authors’ hospital with chronic wounds, an osteomyelitis of the calcaneus, a distal femoral fracture, and multiple episodes of septicemia. On this occasion, she was admitted to the hospital with a grade I open tibia fracture after a fall from a small height. Routine plain radiographs showed a dislocated tibia and fibula fracture (Figure 1). She was admitted, and because of her known neuropathy and problems with tissue and fracture healing in the past, an open reduction and internal fixation with plate osteosynthesis as well as soft tissue coverage combined with an external fixateur and cast therapy were performed to maintain maximal stability, allowing the possibility of full weight-bearing in the post-operative period (Figures 2a and 2b). To prevent full weight-bearing, a wheelchair was ordered and the family was instructed regarding her post-operative care.

Prophylactic antibiotic therapy with cefuroxime was prescribed for seven days. As expected, the girl’s disease – that is, her hyperactivity and her habit of permanently shifting her leg to maintain balance – prevented her from following the post-operative instructions. Because of a broken cast and the consequent instability, the primary cast had to be restored four days after the operation.

**Figure 3:**
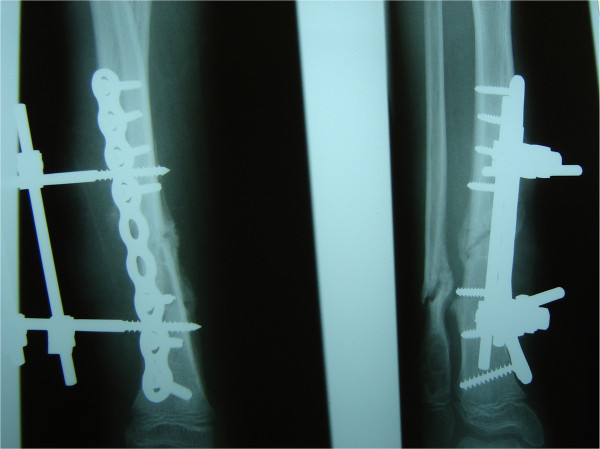
(a,b) Infected non-union of the fracture three months after primary osteosynthesis (anterior-posterior and axial).

**Figure 4:**
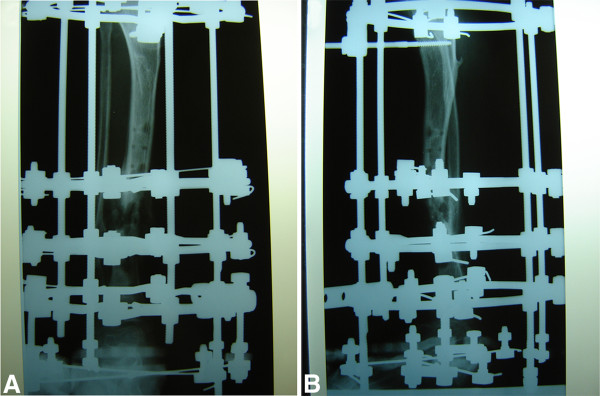
(a,b) Post-operative plain radiographs with the Ilizarov fixateur of the lower extremity (anterior-posterior and axial).

The remainder of her recovery was uneventful, and she was discharged without signs of infection. However, her care was complicated by her continual manipulation of the wound, such as scratching and bouncing at the external fixateur. Extensive wound dressing and coverage of the external fixateur with cotton wool did not fully protect the wound. As expected, she was re-admitted three months later with a wound infection. Plain radiographs showed a mal-union of the tibia fracture (Figures 3a and 3b), and microbiological analysis confirmed an infection with cefuroxime-sensitive *Staphylococcus aureus*. Intravenous antibiotic therapy with cefuroxime was initiated. Because of the mal-union, the plate osteosynthesis was exchanged for an Ilizarov external fixateur (Figures 4a and 4b), which allowed full weight-bearing of the extremity and ensured the stability of the fracture. After surgery, a pin infection of the external fixateur with a local subcutaneous abscess was observed and wound debridement with implantation of a gentamycin-impregnated sponge was undertaken. The antibiotic therapy was continued for six weeks.

**Figure 5:**
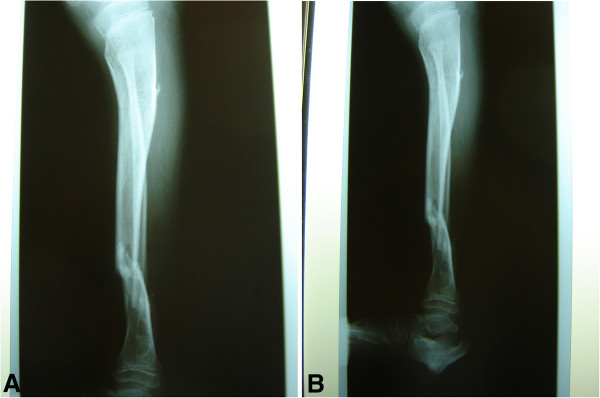
(a,b) Consolidated fracture of the lower extremity four months after primary operation (anterior-posterior and axial) and an incidental finding of a large bone cyst at the distal fibular metaphysis.

Four months after the primary operation, she was re-admitted with loosening of the pins and local signs of an infection. Plain radiographs showed a consolidated tibia fracture without malpositioning of the lower extremity (Figures 5a and 5b), and the external Ilizarov fixateur was removed and exchanged for a cast of the lower extremity for three weeks. The subsequent recovery was uneventful, and wound healing was normal without signs of infection.

## Discussion

Fractures of the lower extremity in childhood are common and many can be treated without surgery, according to the grade of dislocation. Feared long-term complications include varus deformity and recurvation; recurvation has the best prognosis for spontaneous correction, but malposition can also correct spontaneously with the continued growth. A varus malposition can be tolerated up to 25°, and recurvation is tolerable up to 20° [9]. Intramedullary nailing is the treatment of choice in cases of complicated and dislocated fractures, whereas the use of an external fixateur is an option for open and complicated fractures [5,10]. Open fractures of the lower extremity have a high incidence of non-union and infection, especially when the tibia is involved [3]. Thus, primary treatment should include debridement in the operating room to remove devitalized tissue and bone [11]. The Gustilo and Anderson classification, the most accepted method of categorizing open fractures, defines three fracture grades [11]. One of the most feared complications of such fractures is an infection of the wound and subsequent non-union, which often leads to multiple revisions and debridements and complex soft tissue coverage with skin grafting or a free flap [12]. Normally, prophylactic antibiotic therapy is required. In complicated diseases, such as the HSMNs, it may be impossible for patients to follow the planned therapeutic strategy. In our case, we combined plate osteosynthesis of the open tibia fracture with external fixation and cast therapy to maintain maximal stability. But even the combination of these procedures did not lead to an uneventful post-operative recovery.

One possible alternative approach could be the direct use of an external fixateur, such as the Ilizarov spatial frame or TSF. Three previous studies have described the use of the TSF system to treat pediatric fractures of the lower extremity. Al-Sayyad [13] was able to treat 10 fractures in nine children with correct anatomical results after primary repositioning in the operating room. The mean wearing time of the TSF was 19 weeks. In 2008, Eidelman and Katzman [14] described a study population of 13 children with nine open fractures of the tibia for whom TSF was used for treatment. In that study, the nine open tibia fractures were first stabilized with a monolateral external fixateur, and after a mean time of 41 days, these were replaced with a TSF. These patients had a mean wearing time of 11 weeks [14]. The third report, by Gessmann *et al*. [4] in 2010, described a case of an open grade III tibia fracture in a child who had massive soft tissue damage and who was first treated with a monolateral external fixateur that was replaced with a TSF after 11 days. The use of the TSF was necessary because the primary treatment had to include and tolerate an angular shortening of the leg of 2.5cm, a recurvation of 7°, and translation to lateral-posterior to close the soft tissue damage. The mean wearing time was 12 weeks [4]. In these studies, many of the patients had massive tissue damage with post-operative malpositioning or shortening of the tibia. Thus, TSF was used as a possible treatment option to correct secondary deformities.

It is important to note that therapy with the Ilizarov spatial frame or TSF system may be complicated by pin infection and extended wear times until fracture healing is completed. In addition, external fixation is associated with less patient comfort and, for children, is often intolerable [15]. In cases of open tibia fractures in adults who have manifest neuropathy, which often is caused by diabetes mellitus, the use of an external fixateur is one treatment option for which good results have been described [16]. The primary use of the Ilizarov fixateur should be discussed in this case because the child had a manifest neuropathy. It is also appropriate to address whether primary stabilization of the fracture with an Ilizarov fixateur, instead of the osteosynthesis combined with an external Arbeitsgemeinshaft für Osteosynthesefragen (AO) fixateur, should have been performed to prevent a non-union. The causes of a non-union in this case may have included (a) the open fracture, which may have been larger than grade I, and a manifest tissue injury; (b) the placement of foreign material (plate, screws, and external fixateur pins), which carries its own risk of infection; and (c) the peripheral neuropathy.

This case highlights the necessity of the salvage procedures available to the orthopedic surgeon for the treatment of post-operative complications. A recommendation for a single best treatment in case of a neuropathy cannot be made, and treatment must be decided individually. A therapy regime in children with neuropathy must include stabilization of the fracture with closure of the soft tissue damage, allowing the possibility of normal wound healing and potential full weight-bearing on the extremity. A standard therapy does not currently exist, and often two or three therapy strategies must be combined. It remains to be determined whether the primary use of an Ilizarov fixateur as the definitive treatment in the case of an open fracture of the tibia combined with a pre-existing neuropathy is the treatment of choice.

## Conclusions

In pediatric patients who have neuropathies and who, owing to their disease, may be unable to follow all post-operative instructions, an open fracture of the lower extremity is a challenge for the orthopedic surgeon, and salvage procedures should be kept in mind in case of complications. Many therapeutic strategies exist: osteosynthesis, intramedullary nailing, cast therapy, or the use of external fixateurs, such as the Ilizarov spatial frame or TSF systems. The therapeutic modality should be decided individually on the basis of which approach may lead to a stabilization of the fracture with closure of the soft tissue damage and may prevent further complications, such as non-union or infection, and thus guarantee the protection of the child. A standard strategy does not currently exist, and a combination of different therapy options may be necessary to achieve acceptable results. The direct use of an external fixateur such as the Ilizarov spatial frame or TSF should be kept in mind as a possible treatment option, especially in a child with pre-existing neurological or endocrine pathologies.

## Consent

Written informed consent was obtained from the patient’s parents for publication of this case report and accompanying images. A copy of the written consent is available for review by the Editor-in-Chief of this journal.

## Abbreviations

HSMN, hereditary motor and sensory neuropathy; TSF, Taylor spatial frame.

## Competing interests

The authors declare that they have no competing interests.

## Authors’ contributions

MG collected the data and was a major contributor to the manuscript. MD analyzed the data regarding the HSMNs. TAS was a contributor to the operative treatment of pediatric fractures. TK analyzed the patient data. All authors read and approved the final manuscript.
